# Novel electrochemical strategies for the microbial conversion of CO_2_ into biomass and volatile fatty acids using a fluid‐like bed electrode in a three‐phase reactor

**DOI:** 10.1111/1751-7915.14383

**Published:** 2024-01-17

**Authors:** María Llorente, Sara Tejedor‐Sanz, Antonio Berná, Carlos Manchón, Abraham Esteve‐Núñez

**Affiliations:** ^1^ Department of Chemical Engineering Universidad de Alcalá Alcalá de Henares Madrid Spain; ^2^ IMDEA WATER Alcalá de Henares Madrid Spain

## Abstract

Microbial electrosynthesis (MES) constitutes a bioelectrochemical process where bacteria uptake electrons extracellularly from a polarized electrode to incorporate them into their anabolic metabolism. However, the efficiency of current MES reactor designs can be lower than expected due to limitations regarding electron transfer and mass transport. One of the most promising bioreactor configurations to overcome these bottlenecks is the Microbial Electrochemical Fluidized Bed Reactor (ME‐FBR). In this study, microbial CO_2_ fixation is investigated for the first time in a ME‐FBR operated as a 3‐phase reactor (solid–liquid–gas). An electroconductive carbon bed, acting as a working electrode, was fluidized with gas and polarized at different potentials (−0.6, −0.8 and −1 V vs. Ag/AgCl) so it could act as an electron donor (biocathode). Under these potentials, CO_2_ fixation and electron transfer were evaluated. Autotrophic electroactive microorganisms from anaerobic wastewater were enriched in a ME‐FBR in the presence of 2‐bromoethanosulfonic acid (BES) to inhibit the growth of methanogens. Cyclic voltammetry analysis revealed interaction between the microorganisms and the cathode. Furthermore, volatile fatty acids like propionate, formate and acetate were detected in the culture supernatant. Acetate production had a maximum rate of ca. 1 g L^−1^ day^−1^. Planktonic cell biomass was produced under continuous culture at values as high as ca. 0.7 g L^−1^ dry weight. Overall, this study demonstrates the feasibility of employing a fluidized electrode with gaseous substrates and electricity as the energy source for generating biomass and carboxylic acids.

## Graphical abstract

Overall, this study demonstrates the feasibility of employing a fluidized electrode with CO_2_ and electricity as the energy source for generating microbial biomass and carboxylic acids. Planktonic cell biomass was produced under continuous culture at values as high as ca. 0.7 g L^−1^ dry weight.

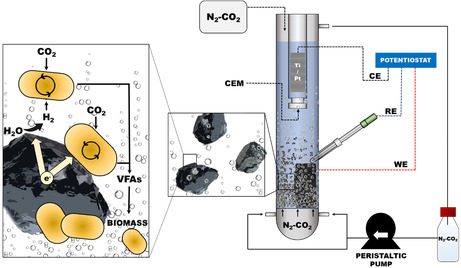

## INTRODUCTION

Since the Paris Agreement, governments have been striving to face and reduce greenhouse gas emissions (United Nations, 2015). Microbial Electrosynthesis (MES) has gained attention in the last decades as a combination of microbial‐catalysed reactions with electrochemical reactions to transform a substance into the desired product (Harnisch & Holtmann, 2017; Schröder et al., 2015). MES for CO_2_ conversion proceeds through several carbon fixation pathways in autotrophic microorganisms so‐called electrotrophs. These pathways include the reductive pentose phosphate cycle, reductive tricarboxylic acid (TCA) cycle and reductive acetyl‐CoA pathway, which is called the Wood‐Ljungdahl (WL) pathway (Lee et al., 2021). Homoacetogenic bacteria, that fix CO_2_ through the Wood‐Ljungdah pathway (Vilanova, 2016), use the electrons provided by the electrode together with CO_2_ to produce Acetyl‐CoA as a precursor for biomass production (Latif et al., 2014; Vassilev et al., 2022) and valuable extracellular multicarbon materials (Zhang et al., 2013) such as organic compounds like acetate (C2) (Bajracharya, 2016; May et al., 2016; Nevin et al., 2011). Furthermore, electrotrophs can also elongate the carbon chain via a reverse β‐oxidation pathway to produce medium‐chain fatty acids (Shen et al., 2018) such as butyrate (Ganigué et al., 2015) or caproate by using the electrons from a cathode together with C2 compounds originated also from cathodic reactions. Ethanol (Blasco‐Gómez et al., 2021) and isobutanol, n‐butanol and n‐hexanol (Vassilev et al., 2018) can be generated via solventogenesis. Sustainable chemicals like bioplastics were also synthesized in MES (Bian et al., 2020; Pepè Sciarria et al., 2018). Thus, the MES is a flexible technology that can be applied to decarbonize and upgrade industrial and wastewater treatment plants, producing a wide array of green and sustainable chemicals (Dessì et al., 2021).

Researchers have studied microorganisms' ability for extracellular electron uptake by electrodes, direct electron transfer (DET). However, microbial electrosynthesis might not depend on the presence of a specialized direct electron uptake system but rather on enzyme‐mediated electron transfer (Deutzmann et al., 2015), or mediators produced electrochemically, such as hydrogen (indirect electron transfer, IET) (Jourdin et al., 2016; Karthikeyan et al., 2019). Indeed, hydrogenotrophic microorganisms are capable of increasing the H_2_ evolution rate on a cathode by maintaining low H_2_ partial pressure by H_2_ consumption (Philips, 2020). These processes have been investigated using different reactor configurations and designs (Bajracharya et al., 2022; Liu et al., 2023). The state of art includes H‐type reactors in which polarization is provided by photovoltaic energy (Nevin et al., 2010), concentric tubular reactors (Batlle‐Vilanova et al., 2017), modular stacked reactors, commonly known as sandwich configuration, (Blasco‐Gómez et al., 2021) three‐chamber MES reactors (Gildemyn et al., 2015), stirred tank reactors (Krieg et al., 2018), photo‐driven MES reactors for culturing purple phototrophic bacteria (PPB) (Manchon et al., 2022), bubble column reactors (Asimakopoulos et al., 2018; Enzmann et al., 2019), packed/fixed and moving bed reactor (Marshall et al., 2013; Vassilev et al., 2019), non‐electroconductive moving bed MES reactor (Cai et al., 2022) or gas diffusion reactors (Bajracharya et al., 2016).

However, MES systems comprise technological challenges pertaining to their productivity and microbe‐material interactions, such as poor gas–liquid mass transfer, low biomass and biofilm coverage on the cathode (Bajracharya et al., 2022) The bioreactor configuration is a crucial parameter in gas fermentation, as it affects gas–liquid mass transfer. These challenges present problems regarding low energy conversion efficiency, making this technology far from the technical and economical solutions of current industrial processes (Prévoteau et al., 2020). Another reactor configuration concern is the oxygen dilemma, whereby oxygen evolution must be separated by a cationic exchange membrane between the cathode and anode chamber (Abdollahi et al., 2022). Single‐chamber, membrane‐less reactors are unsuitable for MES because the oxygen produced at the anode can inhibit the strictly anaerobic microorganisms in the cathode chamber (Liu et al., 2023) and can be reduced by abiotic or biotic cathode reactions and thus decrease the coulombic efficiency (CE) (Dessì et al., 2021; Giddings et al., 2015).

In this particular scenario for finding a suitable bioelectrochemical configuration a new type of bioreactor, the Microbial Electrochemical Fluidized Bed Reactor (ME‐FBR) may offer features to overcome some MES limitations. ME‐FBR originally demonstrated that electroactive microorganisms like those from *Geobacter* genus could grow under a planktonic state by performing direct extracellular electron transfer to a fluid‐like electrode (Tejedor‐Sanz et al., 2017). The combination of a gas column reactor and a bioelectrochemical reactor should address the main problems in the MES reactors studied thus far (Bajracharya et al., 2022). Both configurations have previously been developed at the pilot and commercial scales. Like the continuously operated bubble‐column/gas‐lift loop reactors to produce ethanol with these technologies of LanzaTech (Takors et al., 2018) and the bioelectrochemical reactor for anodic electrobioremediation of industrial wastewater (Asensio, Llorente, Fernández, et al., 2021; Asensio, Llorente, Tejedor‐Sanz, et al., 2021; Tejedor‐Sanz et al., 2018, 2019). In this reactor configuration, the electrode must have a highly biocompatible surface, high chemical stability, high electron transfer rate and high hydrophilicity (Noori et al., 2020). Interestingly, selecting the bed material (electroconductive granular material like carbon or graphite‐based materials) may allow the co‐growth of biofilm and planktonic cells (Tejedor‐Sanz et al., 2019). The fluidization avoids clogging or channelling/tunnelling due to the lack of dense packing of bed material and subsequent biofilm growth. Also, fluidized bed electrodes moved by gas solve the low solubility of CO_2_/H_2_ in the fermentation medium (Bajracharya et al., 2022). Furthermore, this mode of operation enhances mass transfer (Tejedor‐Sanz et al., 2017). The fact that such a fluid‐like electrode has been successfully validated as a biocathode for reducing nitrate (Tejedor‐Sanz et al., 2020) suggests a potential use to support reductive microbial metabolism like those after bioelectrosynthesis.

Thus, the current work explores MES in a CO_2_‐fixing context with a ME‐FBR to generate cell biomass and organic acids. The ME‐FBR configuration integrates, for the first time, a two‐chamber configuration to separate a fluid‐like biocathode from the counter electrode while using a gas phase to fluidize the electroconductive bed.

## EXPERIMENTAL PROCEDURES

### Bacterial growing conditions

A mix of activated sludge and anaerobic digester sludge, taken from an urban wastewater treatment plant (WWTP) located in Guadalajara (Spain), was used as microbial inoculum (Anwer et al., 2021). The medium to select and enrich chemolithoautotrophic bacteria was freshwater medium (FWM) NaHCO_3_ 2.5 g L^−1^, NH_4_Cl 0.5 g L^−1^, NaH_2_PO_4_·2H_2_O 0.41 g L^−1^, KCl 0.1 g L^−1^, a mixed of vitamins 10 mL L^−1^, a mixed of minerals 10 mL L^−1^ (Tejedor‐Sanz et al., 2017) and adjusted to pH 6 (pHmeter Crison pH 25). 2 g L^−1^ of 2‐bromoethanosulfonic acid (BES) (Acros Organics) was added to avoid the growth of methanogens (May et al., 2016; Tharak & Venkata Mohan, 2021). The medium was divided into 100 mL serum bottles, sealed with a butyl septum and bubbled with H_2_:CO_2_ (70:30 v/v) at 30°C. The serum bottles, containing 50 mL of medium, were inoculated with the activated sludge. 50 mL of the enriched chemolithoautotrophic microbial community was used as inoculum for the ME‐FBR. The biomass was taken from the medium by centrifugation (10 min at 9500 rpm, 25°C in Multifuge 3 L‐R, Heraeus) and resuspending the pellet in 50 mL of the freshwater medium inside an anoxic chamber (Coy, US). Then, the cell suspension was used to inoculate the ME‐FBR that contained ca. 70 mL of FWM. A mixed culture was selected after several transfers before inoculating both batch or continuous reactors.

### Microbial Electrochemical Fluidized bed reactor: design and construction

**FIGURE 1:**
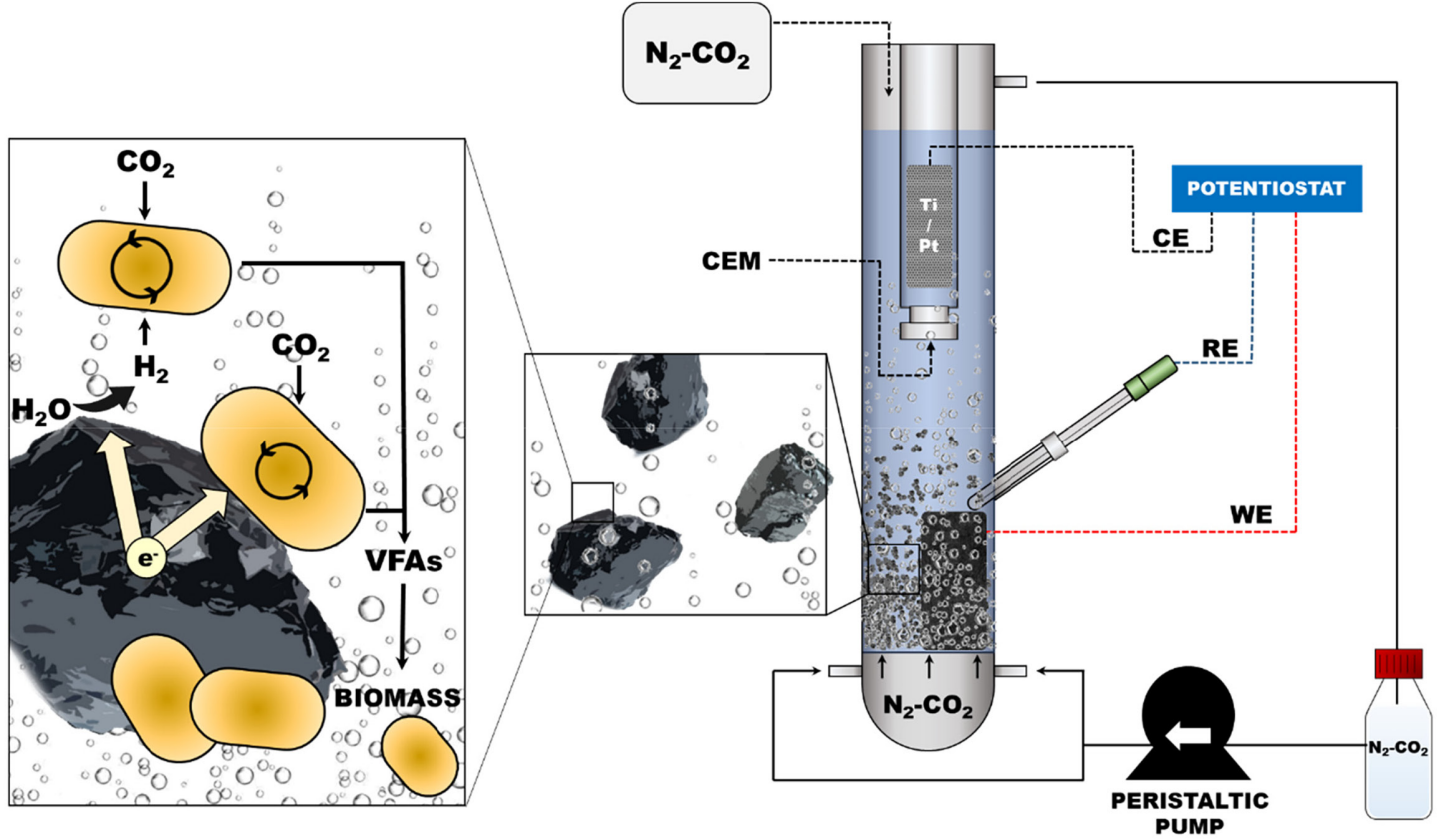
Scheme of the Microbial Electrochemical Fluidized Bed Reactor setup and hypothetical electrochemical/biological reactions. CE, counter electrode; CEM, cation exchange membrane; RE, reference electrode; VFAs, volatile fatty acids; WE, working electrode.

The ME‐FBR consisted of a tubular reactor of 30 cm height and 3 cm inner diameter (ID) (main chamber) with a rounded bottom. The top of the ME‐FBR was sealed to maintain an anoxic environment with a rubber cap. A glassy porous plate (porous diameter 2, 40–100 μm) was placed in the column at ca. 7 cm from the bottom to distribute the gas flow fed in the main chamber, forming small diameter gas bubbles that created a larger gas–liquid interface (Campani et al., 2015). A graphite paper (100 × 25 × 0.1 mm, Mersen) was the current collector. In the main chamber was placed a second smaller tubular chamber made of glass containing a cationic exchange membrane of 15 mm diameter (CEM, Nafion) (Figure 1). This chamber contained a dimensionally stable anode (DSA, Magneto) made of a Ti mesh coated with Pt immersed in 20 mL of FWM. This medium was replenished every 2–3 days due to evaporation. Over the porous plate, in the main chamber, a bed of glassy carbon (GC) particles (size 0.2–0.4 mm diameter Sigradur G, HTW, Germany) was placed that would act as a fluidized bed electrode (cathode). The glassy carbon particles do not present porosity as determined by the helium permeability by the vacuum drop method (10^−11^ cm^2^ s^−1^), and the average surface area of the total amount of particles is 0.168 m^2^ calculated with ImageJ (SI). The main chamber volume was 120 mL, from the porous plate to the top of the column, including the fluidized bed volume (cathode, 12.5 mL). The net reactor volume (NRV) was 120 mL during the experiment with and without the fluidized bed particles. The bed, when present, was 4% of the total volume.

A reference electrode of Ag/AgCl 3 M KCl (Xylem) was placed in the cathodic chamber close to the fluidized bed cathode.

### Operational conditions

#### Phase 1

For the first characterization of the system, the reactor was operated for 25 days in batch mode and sparged with a continuous flow of N_2_:CO_2_ (80:20) in the absence of a glassy carbon bed to evaluate the role of only the graphite paper as the current collector. The current collector was polarized at −0.6 V. Then the potential was shifted to −0.8 and −1 V versus Ag/AgCl gradually to promote hydrogen production and eventually stimulate microbial growth and fatty acids production by acidogenic microorganisms (Blanchet et al., 2015). Samples were taken daily from the reactor to measure pH, optical density (OD 600 nm) and volatile fatty acids concentration by HPLC. The medium was replenished periodically and the reactor was re‐inoculated to provide fresh cells for the process.

#### Phase 2

After that period, the bed of glassy carbon particles (12.5 mL) was used as a biocathode, and the ME‐FBR was operated with graphite paper as the current collector. A peristaltic pump (Heidolph 5001) recirculated the gas from the headspace to the bottom to fluidize the glassy carbon bed. Gas circulated through a 1 L Pyrex bottle filled with N_2_:CO_2_ (80:20) to avoid recirculating liquid into the reactor. The gas first fluxed through the porous glassy plate and then expanded the fluidized bed (Figure S1). The pump recirculated the gas at 0.35 L min^−1^ with a linear velocity of 0.84 cm s^−1^ (calculated from the column section and the gas flow). A sealed bag filled with N_2_:CO_2_ (80:20) was connected to the upper outlet of the reactor to ensure anaerobic conditions and C source inside the reactor (Figure 1). The reactor was operated in batch mode, and the medium was replaced every week for ca. 3 months coupled to the inoculation of the reactor.

#### Phase 3

After that, the reactor was operated in continuous mode by feeding fresh medium with a peristaltic pump (Watson Marlow 205S) at a hydraulic retention time (HRT) of 41 h (Figure S2). To restart the operation of the reactor, the medium was replenished and the reactor inoculated. The influent (FWM described above) was continuously sparged with N_2_:CO_2_ (80:20). Cysteine 0.5 mM and resazurin 4 μM were added as an O_2_ scavenger and a redox indicator, respectively (Table 1). After 2 weeks of operation, the HRT decreased from 41 to 20 h. An abiotic control was performed to study the electrochemical behaviour of the resazurin in the system. (Figure S3).

**TABLE 1:** Operational procedures of the experiment.

	Current collector	Fluidized bed	Feeding regimes	Polarization (vs. Ag/AgCl)	Compounds added	Gas stream
Phase 1	Graphite paper	—	Batch	−0.6 V −0.8 V −1 V	BES	N_2_:CO_2_ (80:20)
Phase 2	Graphite paper	Glassy carbon	Batch	−1 V	BES	Gas recirculation
Phase 3	Graphite paper	Glassy carbon	Continuous	−1 V	BES Cysteine Resazurin	Gas recirculation

*Note*: BES stands for 2‐bromoethanosulfonic acid a methanogens inhibitor.

### Electrochemical measurements

The cathode was polarized at −0.6, −0.8 and −1 V versus an Ag/AgCl electrode. The electrochemical analysis was performed by a potentiostat (Nanoelectra NEV 3, compliance ±9 V). Cyclic voltammograms were performed from −0.9 to 0.3 V versus Ag/AgCl and at a scan rate of 0.005 V s^−1^, current consumption was divided by the net reactor volume (NRV), 120 mL, to calculate current density.

### Analytical methods

An HPLC series 1100 (Agilent) high‐pressure liquid chromatograph equipped with a UV detector (210 nm) and a Supelco C‐610H column determined the volatile fatty acids concentrations. The HPLC methodology was used to analyse formate, acetate, propionate, butyrate, succinate and fumarate and the respective calibration curves were performed with 100 mM, 50 mM, 25 mM, 12.5 mM, 6.25 mM, 3.125 mM and including an analyte‐free solution. 0.1% H_3_PO_4_ was used as the mobile phase at a flow rate of 0.5 mL min^−1^.

### Microscopy analysis

A scanning electron microscope (SEM) was used to study the microbial colonization of the graphite paper for the experimental *Phase 1*. The current collector was extracted by removing the rubber stopper with a continuous flow of N_2_‐CO_2_ before adding the conductive particles, cutting a small piece off and reintroducing the current collector into the reactor. The small sample and control were rinsed with distilled water to remove the planktonic microorganisms. Samples for the SEM were fixed with 5% (v/v) glutaraldehyde in cacodylate buffer (0.2 M, pH 7.2) and gradually dehydrated with ethanol solutions (25%, 50%, 70%, 90% and 100%, 10 min each step). Subsequently, samples were washed twice with acetone for 10 min each and immersed in anhydrous acetone at 4°C overnight. Finally, the samples were dried in CO_2_ at the critical point and coated with gold. Micrographs were taken using a scanning electron microscope DSM‐950 (Zeiss).

### Spectrophotometry

Optical density (600 nm) was used to measure the planktonic microbial growth in the reactor with the Spectrophotometer (UV‐1800, Shimadzu, UV‐Spectrophotometer). The optical density was interpolated to volatile suspended solids with a calibration curve.

### Volatile suspended solids VSS

The VSS concentration (gVSS L^−1^) was measured according to standard methods (Eaton et al., 2005). A calibration curve with OD (600 nm) and VSS obtained this equation *y* = 807.88*x* (*R* ^2^ = 0.95), corresponding x and y to OD and VSS, respectively. Volatile suspended solids are also referred as dry weight and planktonic biomass.

### Coulombic efficiency (CE)

The efficiency of capturing electrons from the electric current to the product was calculated using (Bajracharya et al., 2017):

$$\mathrm{CE}\;\left(\%\right)=\frac{F\times \mathrm{\Delta N}\times f}{\int \mathrm{\Delta I}\;\mathrm{dt}}\times 100$$

where F is Faraday's constant (96.485 C mol^−1^). The CE were calculated separately for acetate and biomass production. CE for acetate production was calculated, considering (i) ΔN is the increment in moles of acetate, (ii) f represents the molar conversion factor of 8 [8 moles of electrons needed to fix 1 mol of acetate (Bajracharya et al., 2017; Patil et al., 2015)], and (iii) I is the current consumed. CE for biomass was calculated, considering (i) ΔN as the increment in moles of biomass, for example, CH_1.8_O_0.38_N_0.18_ (Gottschalk, 1986; Carlozzi & Sacchi, 2001; McKinlay & Harwood, 2010), (ii) f as the molar conversion factor is 4.5, (4.5 moles of electrons are needed to fix 1 mole of biomass) (McKinlay & Harwood, 2010) and (iii) I is the current consumed associated to the biomass produced.

## RESULTS AND DISCUSSION

After the initial enrichment of a chemolithoautotrophic microbial community and an initial inoculation in a ME‐FBR, the bioelectrochemical activity was characterized by employing different electrochemical and feeding operational conditions. As a benchmark, the system's performance was first characterized by utilizing only the current collector (*Phase 1*) and then operated with the fluid‐like cathode under different feeding regimes (*Phase 2* and *3*).

### Starting up: CO_2_ Fixation using a conventional graphite electrode (*phase 1*)

**FIGURE 2:**
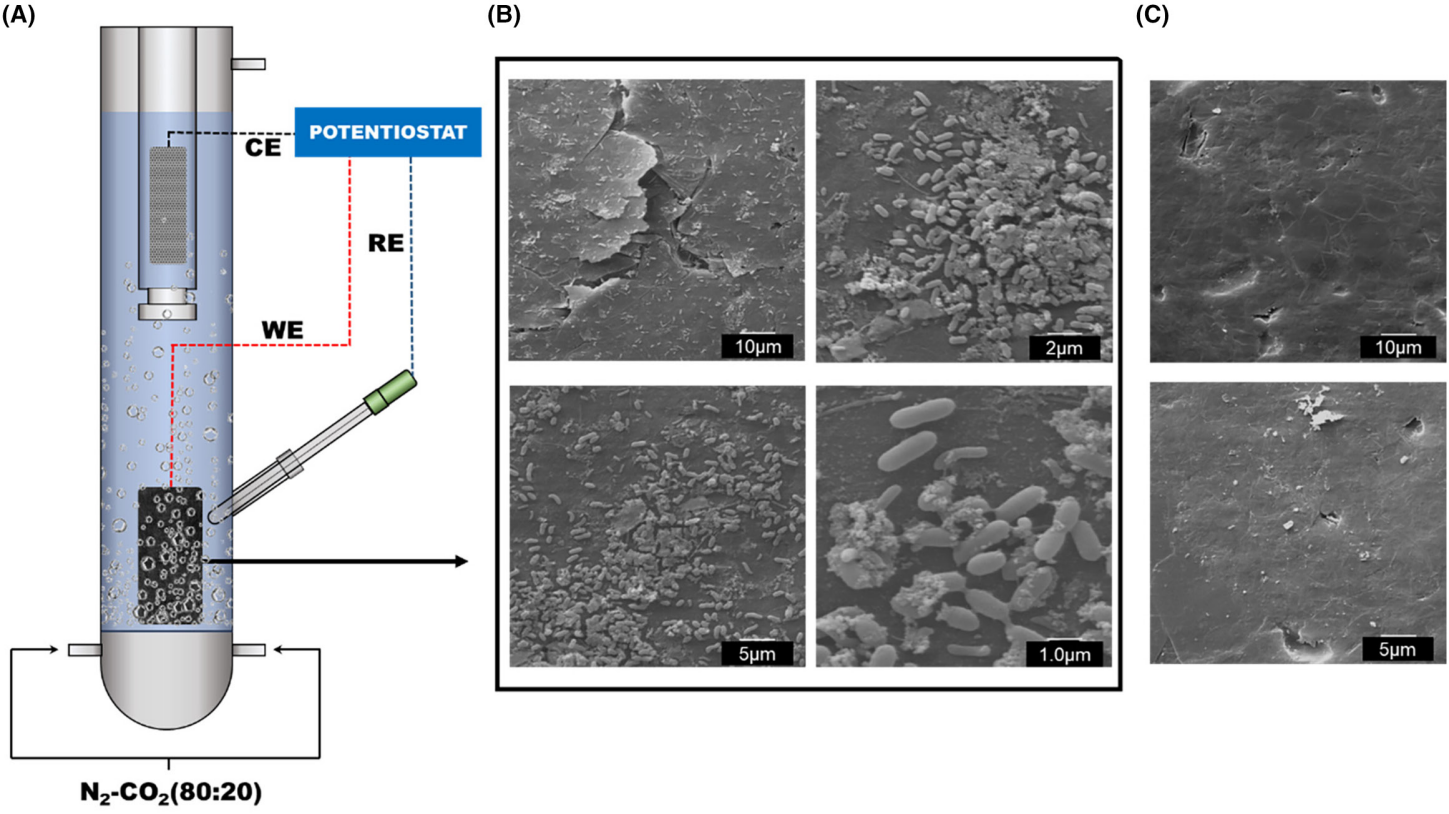
Bioreactor operation during *phase 1* (batch mode) using the solely current collector as the cathode. (A) Schematic of the operational setup. (B) SEM images of the current collector polarized at −1 V versus Ag/AgCl at different magnifications. (C) SEM images of the control, abiotic current collector.

**FIGURE 3:**
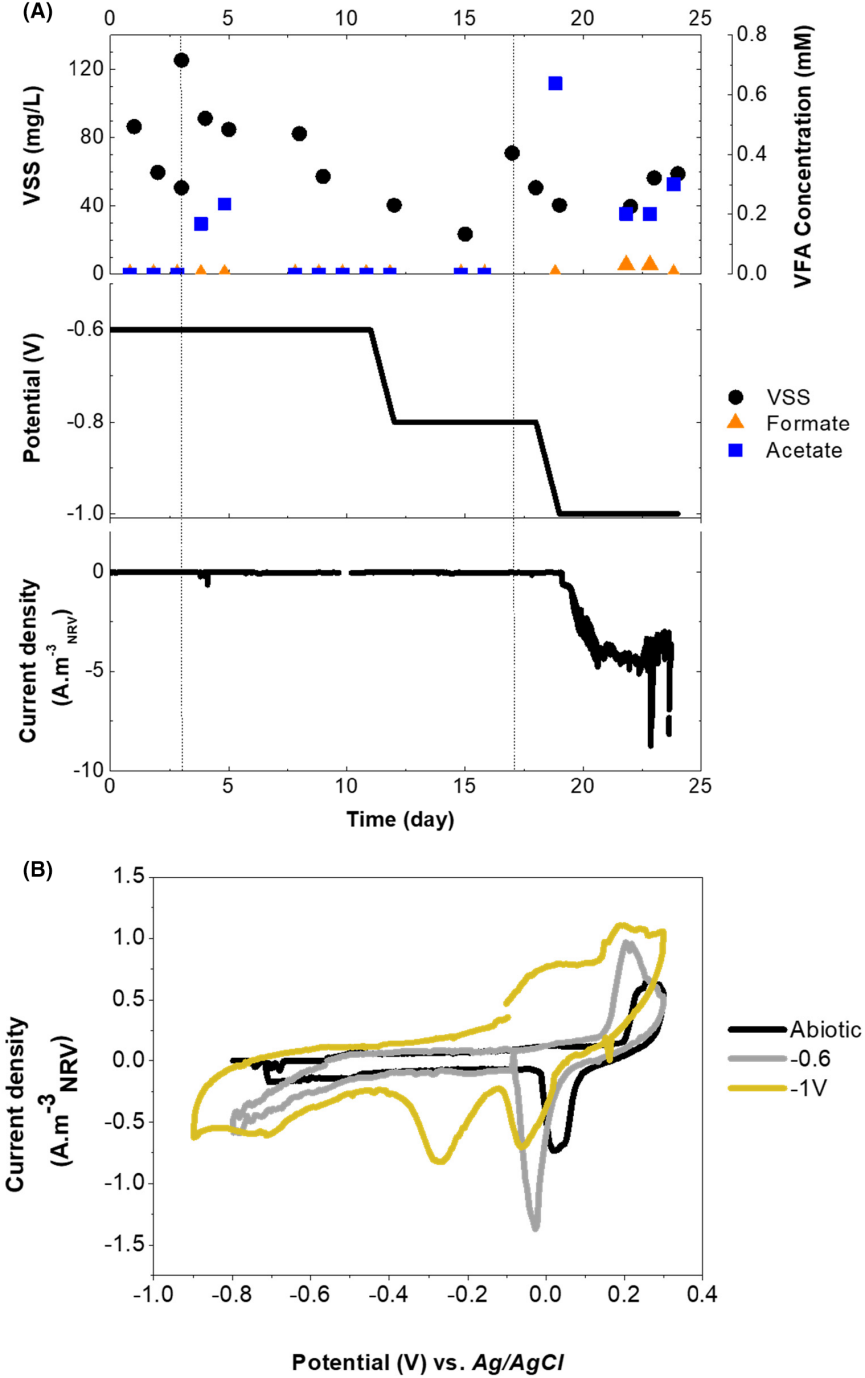
Reactor operation using the current collector solely as the cathode. (A) Volatile suspended solids (VSS), volatile fatty acids concentration, the potential applied to the current collector and current density consumed by the system. Dotted lines indicate medium replenishment and inoculation of the reactor. (B) Cyclic voltammograms of the system under abiotic and biotic conditions, with the current collector polarized at −0.6 and −1 V versus Ag/AgCl. NRV, net reactor volume.

Initially, the current collector was studied to determine the role of using CO_2_ as a terminal electron acceptor. The two‐chamber configuration's capacity to convert CO_2_ to products was evaluated using only graphite paper as the cathode (working electrode), (*Phase 1*, Figure 2A). This graphite paper will eventually act as the current collector in the full fluid‐like version of ME‐FBR. The electrical current, biomass production, VFA production and cyclic voltammetry analysis were performed at different cathode potentials, −0.6, −0.8 and −1 V, versus Ag/AgCl 3 M KCl. When the system was polarized at −0.6 V versus Ag/AgCl, revealed a maximum acetate production of 0.082 g L^−1^ day^−1^, likely generated by inoculum leftover since current consumption was absent. Polarizing to −0.8 V versus Ag/AgCl, no acetate production was detected and neither was current consumption. For both polarizations, planktonic biomass, measured as VSS, decreased by 12.9 mg L^−1^ day^−1^. When the current collector was polarized to −1 V versus Ag/AgCl acetate increased to 0.31 g L^−1^ day^−1^ (CE 0.5%) by day 19, formate to 0.012 g L^−1^ day^−1^ and planktonic biomass production increased to 3.8 mg L^−1^ day^−1^, achieving a maximum value of 59 mg L^−1^. This level of planktonic biomass production meant a CE of 20% (Figure 3A). Chronoamperometry analysis at −1 V revealed a current consumption increase (day 17), achieving values of −5 A m^−3^_NRV_ (−0.12 A m^−2^ of graphite paper surface), possibly related to the electrochemical production of hydrogen at such low potentials. Such hydrogen production may led to an increase in cell growth and acetic acid concentration, although the last statement should be experimentally demonstrated.

SEM micrographs taken when the cathode was polarized at −1 V versus Ag/AgCl identified bacilli‐shaped bacteria attached to the graphite paper (Figure 2B). Cyclic voltammetry was used to study both hydrogen production and the redox interactions between electroactive microorganisms and the polarized current collector, working as the cathode. Voltammograms were obtained before the system was inoculated (abiotic control) and after inoculation under different polarizations (Figure 3B). The voltammogram under abiotic conditions revealed a reduction peak at approx. 0.02 V versus Ag/AgCl. A reduction peak, centred at lower potentials, was also found in the voltammogram performed after operating the ME‐FBR at −0.6 (grey line) and −1 V versus Ag/AgCl (yellow line), indicating the physicochemical properties of the current collector (Kim, 2011; Pereira et al., 2019) or impurities present on the graphite paper surface. The starting pH under anaerobic conditions was 7 for the abiotic control (Kong et al., 2020). Under polarized conditions, the pH shifted to 7.15 at −0.6 V versus Ag/AgCl and 7.5 at −1 V versus Ag/AgCl. Such an increase in pH means that hydrogen that protons flow from the anodic chamber to the cathodic one through the CEM, may be limited by the membrane size. This mild shift in pH may have contributed to the shifting peak in the voltammograms from the abiotic to lower potentials (Figure 3B). However, an increase in the current consumption (from 0 to −0.5 A m^−3^ at potentials of −0.5 V vs. Ag/AgCl) occurred just in the presence of microorganisms growing at −0.6 V versus Ag/AgCl. In contrast, no new redox centre was observed when the cathode was polarized at −0.6 V versus Ag/AgCl, while the voltammogram obtained when the cathode was polarized at −1 V versus Ag/AgCl showed a new reduction peak at −0.3 V versus Ag/AgCl. Furthermore, a higher capacity likely due to biofilm (Figure 2B) and planktonic growth on the cathode and in the system was observed when the system was polarized to −1 V versus Ag/AgCl. Overall, these differences found between the abiotic and biotic conditions indicate the presence of a redox interaction between the graphite electrode and the microbial community present in the reactor.

### Upgrading CO_2_ fixation using a fluid‐like electrode in batch mode (*phase 2*)

**FIGURE 4:**
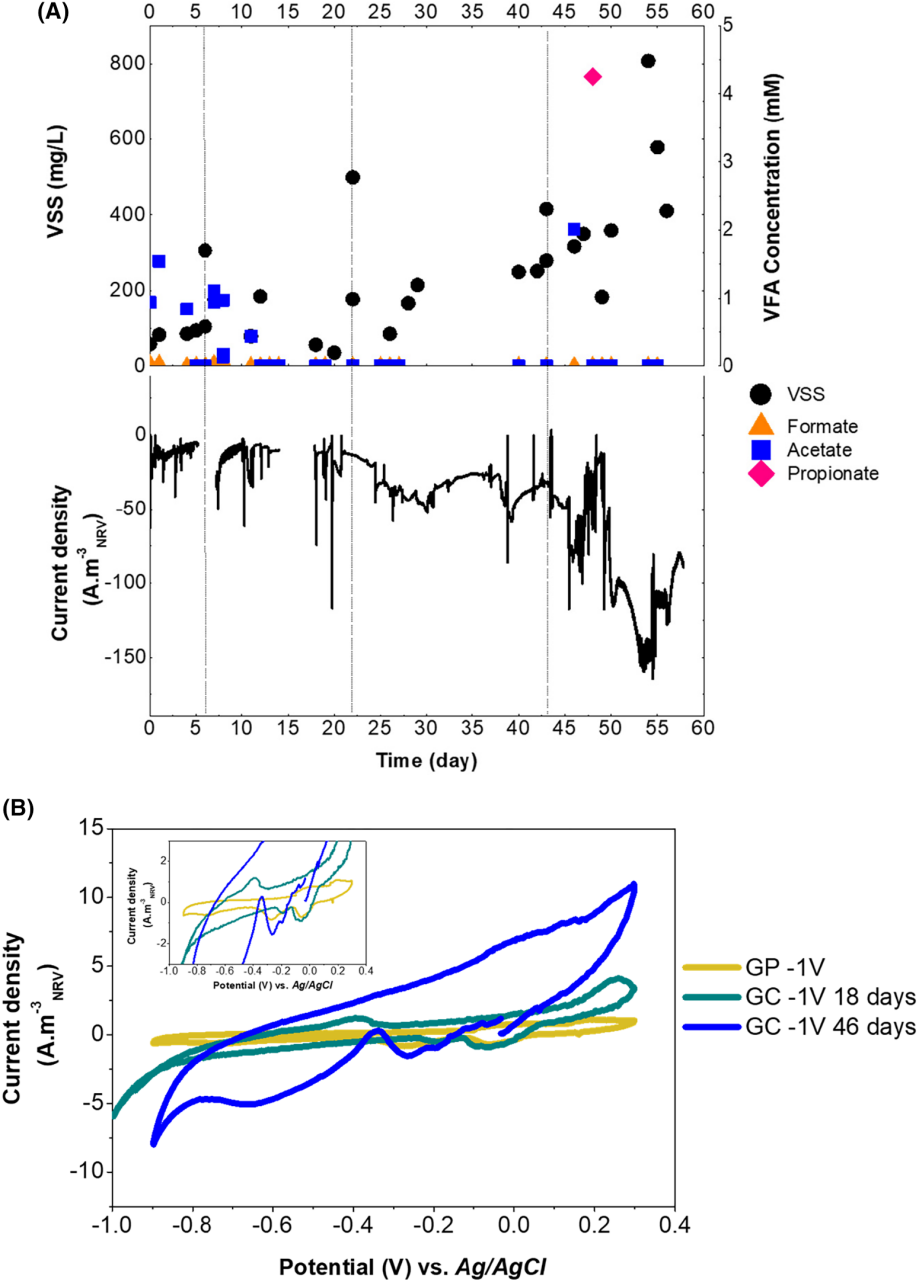
Bioreactor operation during batch mode (*phase 2*), using a fluid‐like cathodic bed polarized at −1 V versus Ag/AgCl. (A) Volatile suspended solids (VSS), volatile fatty acids concentration and current density. Dashed lines indicate media replenishment and inoculation of the reactor. (B) Cyclic voltammograms of the system comparing the performance of the current collector, graphite paper (GP, yellow line) and the fluid‐like glassy carbon electrode (GC) polarized at −1 V versus Ag/AgCl after 18 days (green line), and 46 days (blue line). NRV stands for net reactor volume.

To increase electron accessibility by microorganisms and to maximize planktonic bacteria interaction with an electrode, a bed of conductive GC particles was added (Tejedor‐Sanz et al., 2017). The GC particles in the reactor thereby acted as a cathode, and the graphite paper's primary purpose was as the current collector. The gas from the headspace was recirculated through the bottom of the reactor to expand the GC bed (Figure S1). This gas stream included H_2_ produced at −1 V versus Ag/AgCl. Since higher electroactivity at −1 V versus Ag/AgCl was previously observed, the fluid‐like cathode was polarized to this potential. The conversion of CO_2_ to products was explored under batch mode using the same methodology as in *Phase 1*. To provide fresh nutrients and maintain metabolic activity, the reactor medium was replenished and inoculated regularly (Figure 4A). In the presence of the fluidized electrode, the current density achieved values of −160 A m^−3^_NRV_ (1536 A m^−3^_bed_) while acetic acid production increased to 0.99 g L^−1^ day^−1^. This rate is similar to the production rate of a fixed bed electrode (1.04 g L^−1^ day^−1^) of a long‐term adapted mixed culture using a granular carbon bed as a cathode with H_2_ production, as previously reported (Marshall et al., 2013). The fluctuation in the acetate concentration is explained by the competitive microbial reactions in MES with mixed cultures (Abdollahi et al., 2022). Cell growth in the bulk, showed an increasing trend with time, 0.0067 g L^−1^ day^−1^, reaching a maximum dry weight of ca. 0.8 g L^−1^ measured as VSS after 50 days (CE 15%). The high biomass, no accumulation of fatty acids and the electrical current consumed suggested heterotrophic, electroautotrophic and/or hydrogenotrophic microbial metabolism in the medium coupled to electrochemical reactions. Thus, the fluidized bed reactor was operating as a system to produce microbial biomass mediated by microbial electrosynthesis using CO_2_ as the initial building blocks.

Cyclic voltammetry was performed when the system was polarized to −1 V versus Ag/AgCl at different operational times (Figure 4B). The presence of a fluid‐like electrode polarized to −1 V versus Ag/AgCl, and a specific microbiome enriched for 46 days, outperformed current density consumption (ca. 8‐fold) in comparison with only the current collector. For instance, after polarizing the current collector at ‐1 V versus Ag/AgCl in *Phase 1*, in the voltammogram, −0.6 A m^−3^_NRV_ were consumed at a potential of −0.6 V versus Ag/AgCl (Figure 4B), while −5 A m^−3^_NRV_ were consumed in the fluidized bed after 46 days of operation polarized at the same potential, −1 V versus Ag/AgCl.

The voltammogram corresponding to the system operated with only the current collector showed lower current density than the voltammograms obtained when the bioreactor was operated with the fluid‐like cathode. In the voltammogram of the fluid‐like cathode, after 46 days of operation, all current density values were higher (in absolute value) for all the potentials tested. In addition, a new reduction peak appeared at a potential of −0.6 V versus Ag/AgCl, which was not observed in previous voltammograms and could be related to a change in the mechanism for redox interaction between microorganisms and the fluidized electrode (Harnisch & Freguia, 2012). In both voltammograms with GC particles, hydrogen onset potential was observed in the reduction peaks at −0.9 to −1 V versus Ag/AgCl.

### Upgrading CO_2_ fixation using a fluid‐like electrode in continuous mode (*phase 3*)

**FIGURE 5:**
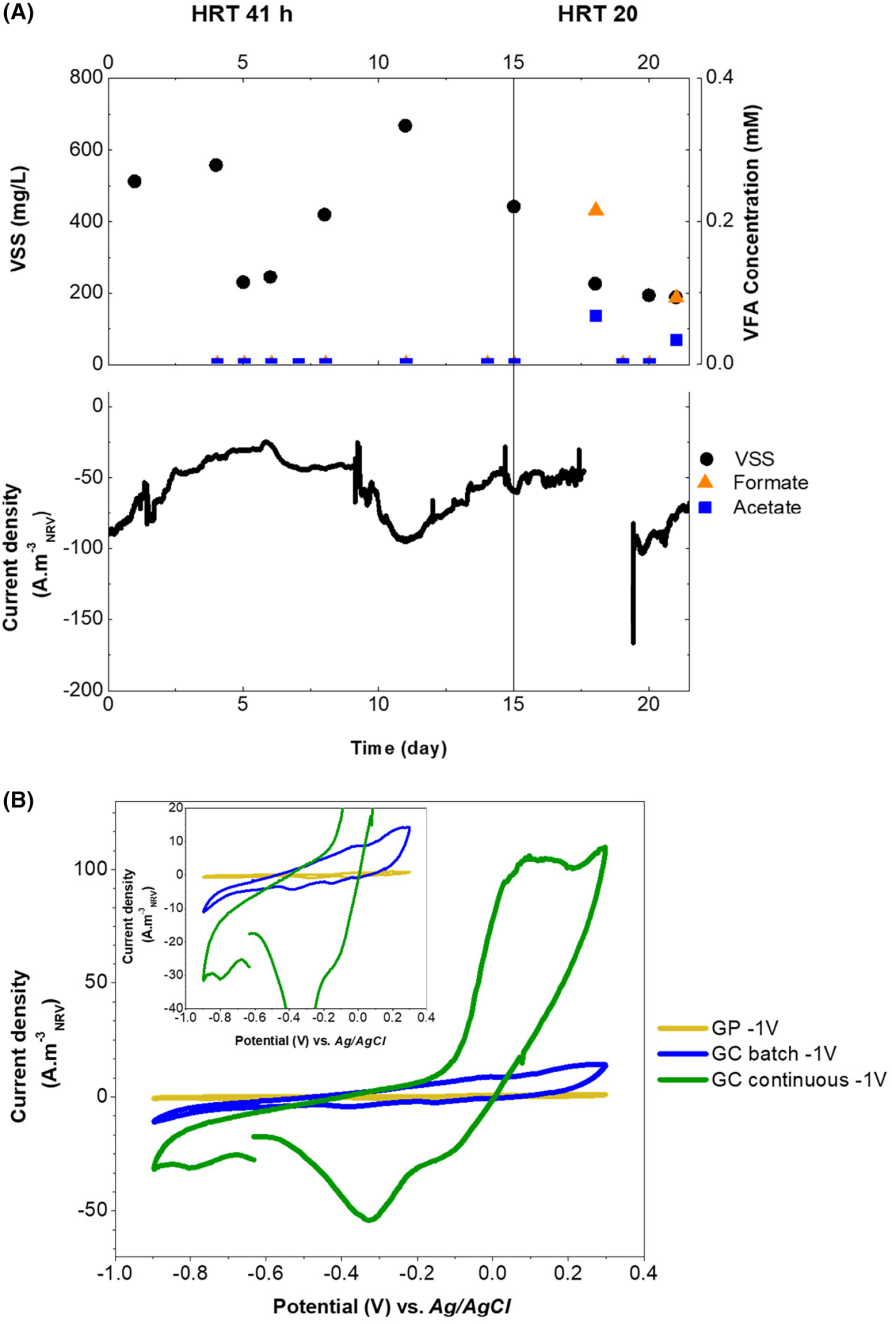
Bioreactor operation during continuous mode (*phase 3*) using a fluid‐like bed as the cathode. (A) Volatile suspended solids (VSS), volatile fatty acids concentration, potentials applied and current density. The vertical line indicates a shift in the HRT. (B) Cyclic voltammetry of the system comparing the performance of the current collector, graphite paper at batch mode (GP, −1 V vs. Ag/AgCl, yellow line), fluid‐like glassy carbon (GC, −1 V vs. Ag/AgCl, blue line) operated in batch mode and fluid‐like glassy carbon operated under continuous mode at −1 V versus Ag/AgCl (green line). NRV, net reactor volume.

The batch operation was followed by a mode of continuous operation for 40 days under two different hydraulic retention times (ca. 41 and 20 h) (Figure S2). Under these operational conditions, acetic and formic acid production was detected, although the production was lower than 0.12 g L^−1^ day^−1^ (Figure 5A). However, the planktonic microbial growth, at −1 V versus Ag/AgCl increased by 0.012 g L^−1^ day^−1^, reaching values of dry weight (VSS) of ca. 0.67 g L^−1^ (CE 11%). After 19 days of operation, the HRT was shifted to 20 h. This decrease in HRT promoted the planktonic cells to be washed out of the reactor (Dürre, 2017) decreasing the biomass production to 0.033 g L^−1^ day^−1^.

Cyclic voltammograms performed in different operational conditions showed remarkable differences (Figure 5B). The voltammogram corresponding to the current collector alone was a straight line without significant peaks. In contrast, the cyclic voltammogram of the fluid‐like electrode polarized at −1 V versus Ag/AgCl under continuous feeding showed a remarkable oxidation peak of 110 A m^−3^_NRV_ and a reduction peak at −50 A m^−3^_NRV_ when the potential was 0.1 V and −0.3 V versus Ag/AgCl respectively. Such value was ca. 10‐fold higher than the highest oxidation and reduction peaks obtained under batch mode conditions. Higher current density values (in absolute value) can be related to higher catalytic activities such as enzyme‐mediated electron transfer, where extracellular redox‐active enzymes, like hydrogenase and formate dehydrogenase, can bind to and interact with the cathodic surface getting reduced (Deutzmann et al., 2015). Thus, ME‐FBR operated in continuous mode host cells under constant division stage avoiding starvation periods or metabolite accumulation, resulting in a higher abundance of electroactive microorganisms or a higher extracellular electron uptake activity enhanced by the culture conditions or hydrogen production. The system presented a start‐up period, related to the primary colonization and growth of the microorganisms, corresponding to the operation of the system in batch mode and a second period where microorganisms are adapted and divided when the system was operated in continuous mode.

Comparing the results obtained for all the phases in the experiment, the phase with major acetate and biomass production was in phase 2, corresponding to the operation of the system under batch mode and with a fluidized bed as biocathode (Table 2).

**TABLE 2:** Operational procedures and main results of the experiment.

	Phase 1	Phase 2	Phase 3
Current collector	Graphite paper	Graphite paper	Graphite paper
Fluidized bed	—	Glassy carbon	Glassy carbon
Feeding regimes	Batch	Batch	Continuous
Polarization (vs. Ag/AgCl)	−0.6 V −0.8 V −1 V	−1 V	−1 V
Compound added	BES	BES	BES Cysteine Resazurin
Gas stream	N_2_:CO_2_ (80:20)	Gas recirculation	Gas recirculation
Catholite pH	7.15 ± 0.16 7.27 ± 0.1 7.5 ± 0.09	7.14 ± 0.36	7.05 ± 0.22
Min. current (A m^−3^_NRV_)	−5	−160	−93.75
Min. current (A m^−2^)	−0.12	−0.11	−0.07
Max. acetate g L^−1^ day^−1^	0.31	0.99	0.12
CE acetate	0.5%	1%	
Max. biomass mg L^−1^	59	800	670
CE biomass	20%	15%	11%

*Note*: BES stands for 2‐bromoethanosulfonic acid a methanogens inhibitor.

## CONCLUSIONS AND FURTHER RECOMMENDATIONS

For the first time, this study demonstrates that bioelectrosynthesis is a feasible process in a 3‐phase ME‐FBR fed with CO_2_ gas as an electron acceptor and carbon source. Our research shows that planktonic biomass was produced in *phase 2* at values as high as 0.8 g L^−1^, while acetate was produced at a rate of 0.99 g L^−1^ day^−1^. This viable system showed clear redox interactions between microorganisms and the fluid‐like cathode.

For further work, a deep study focused on long‐chain carbon metabolites to elucidate what reactions occur in the ME‐FBR. Specifically, studying the complete carbon balance under different operation conditions (electrode potential, HRT, pH, etc.) and characterization of the microbial communities involved.

## AUTHOR CONTRIBUTIONS

**María Llorente:** Investigation (equal); writing – original draft (equal). **Sara Tejedor‐Sanz:** Investigation (equal); methodology (equal). **Antonio Berná:** Formal analysis (equal). **Carlos Manchón:** Conceptualization (equal); data curation (equal); formal analysis (equal). **Abraham Esteve‐Núñez:** Conceptualization (equal); supervision (equal); writing – review and editing (equal).

## FUNDING INFORMATION

This work was funded by i) Grant RTI2018‐101974‐B‐C‐21 funded by MCIN/AEI/ 10.13039/501100011033, ii) Grant P2018/emt‐4341 funded by Regional Government of Madrid, and iii) Grant TED2021‐132870B100 funded by MCIN/AEI/ 10.13039/501100011033 and by the “European Union NextGenerationEU/PRTR”.

## CONFLICT OF INTEREST STATEMENT

The authors declare that they have no known competing financial interests or personal relationships that could have appeared to influence the work reported in this paper.

## Supporting information


Figure S1.

## References

[mbt214383-bib-0001] Abdollahi, M. , Al Sbei, S. , Rosenbaum, M.A. & Harnisch, F. (2022) The oxygen dilemma: the challenge of the anode reaction for microbial electrosynthesis from CO_2_ . Frontiers in Microbiology, 13, 947550. Available from: 10.3389/fmicb.2022.947550 35992647 PMC9381829

[mbt214383-bib-0002] Anwer, A.H. , Khan, N. , Khan, M.D. , Shakeel, S. & Khan, M.Z. (2021) Redox mediators as cathode catalyst to boost the microbial electro‐synthesis of biofuel product from carbon dioxide. Fuel, 302, 121124. Available from: 10.1016/j.fuel.2021.121124

[mbt214383-bib-0003] Asensio, Y. , Llorente, M. , Fernández, P. , Tejedor‐Sanz, S. , Ortiz, J.M. , Ciriza, J.F. et al. (2021) Upgrading fluidized bed bioelectrochemical reactors for treating brewery wastewater by using a fluid‐like electrode. Chemical Engineering Journal, 406, 127103. Available from: 10.1016/j.cej.2020.127103

[mbt214383-bib-0004] Asensio, Y. , Llorente, M. , Tejedor‐Sanz, S. , Fernández‐Labrador, P. , Manchon, C. , Ortiz, J.M. et al. (2021) Microbial electrochemical fluidized bed reactor (ME‐FBR): an energy‐efficient advanced solution for treating real brewery wastewater with different initial organic loading rates. Journal of Environmental Chemical Engineering, 9(6), 106619. Available from: 10.1016/j.jece.2021.106619

[mbt214383-bib-0005] Asimakopoulos, K. , Gavala, H.N. & Skiadas, I.V. (2018) Reactor systems for syngas fermentation processes: a review. Chemical Engineering Journal, 348, 732–744. Available from: 10.1016/j.cej.2018.05.003

[mbt214383-bib-0006] Bajracharya, S. (2016) *Microbial Electrosynthesis of Biochemicals: innovations on biocatalysts, electrodes and ion‐exchange for CO2 supply, chemicals production and separation*. [internal PhD, WU, Wageningen University]. Wageningen University. Available from: 10.18174/385426

[mbt214383-bib-0007] Bajracharya, S. , Krige, A. , Matsakas, L. , Rova, U. & Christakopoulos, P. (2022) Advances in cathode designs and reactor configurations of microbial electrosynthesis systems to facilitate gas electro‐fermentation. Bioresource Technology, 354, 127178. Available from: 10.1016/j.biortech.2022.127178 35436538

[mbt214383-bib-0008] Bajracharya, S. , Vanbroekhoven, K. , Buisman, C.J.N. , Pant, D. & Strik, D.P.B.T.B. (2016) Application of gas diffusion biocathode in microbial electrosynthesis from carbon dioxide. Environmental Science and Pollution Research, 23(22), 22292–22308. Available from: 10.1007/s11356-016-7196-x 27436381

[mbt214383-bib-0009] Bajracharya, S. , Yuliasni, R. , Vanbroekhoven, K. , Buisman, C.J.N. , Strik, D.P.B.T.B. & Pant, D. (2017) Long‐term operation of microbial electrosynthesis cell reducing CO_2_ to multi‐carbon chemicals with a mixed culture avoiding methanogenesis. Bioelectrochemistry, 113, 26–34. Available from: 10.1016/j.bioelechem.2016.09.001 27631151

[mbt214383-bib-0010] Batlle‐Vilanova, P. , Ganigué, R. , Ramió‐Pujol, S. , Bañeras, L. , Jiménez, G. , Hidalgo, M. et al. (2017) Microbial electrosynthesis of butyrate from carbon dioxide: production and extraction. Bioelectrochemistry, 117, 57–64. Available from: 10.1016/j.bioelechem.2017.06.004 28633067

[mbt214383-bib-0011] Bian, B. , Bajracharya, S. , Xu, J. , Pant, D. & Saikaly, P.E. (2020) Microbial electrosynthesis from CO_2_: challenges, opportunities and perspectives in the context of circular bioeconomy. Bioresource Technology, 302, 122863. Available from: 10.1016/j.biortech.2020.122863 32019708

[mbt214383-bib-0012] Blanchet, E. , Duquenne, F. , Rafrafi, Y. , Etcheverry, L. , Erable, B. & Bergel, A. (2015) Importance of the hydrogen route in up‐scaling electrosynthesis for microbial CO_2_ reduction. Energy and Environmental Science, 8(12), 3731–3744. Available from: 10.1039/c5ee03088a

[mbt214383-bib-0013] Blasco‐Gómez, R. , Romans‐Casas, M. , Bolognesi, S. , Perona‐Vico, E. , Colprim, J. , Bañeras, L. et al. (2021) Steering bio‐electro recycling of carbon dioxide towards target compounds through novel inoculation and feeding strategies. Journal of Environmental Chemical Engineering, 9(4), 105549. Available from: 10.1016/j.jece.2021.105549

[mbt214383-bib-0014] Cai, W. , Cui, K. , Liu, Z. , Jin, X. , Chen, Q. , Guo, K. et al. (2022) An electrolytic‐hydrogen‐fed moving bed biofilm reactor for efficient microbial electrosynthesis of methane from CO_2_ . Chemical Engineering Journal, 428, 132093. Available from: 10.1016/j.cej.2021.132093

[mbt214383-bib-0015] Campani, G. , Ribeiro, M.P.A. , Horta, A.C.L. , Giordano, R.C. , Badino, A.C. & Zangirolami, T.C. (2015) Oxygen transfer in a pressurized airlift bioreactor. Bioprocess and Biosystems Engineering, 38(8), 1559–1567. Available from: 10.1007/s00449-015-1397-4 25903476

[mbt214383-bib-0016] Carlozzi, P. & Sacchi, A. (2001) Biomass production and studies on *Rhodopseudomonas palustris* grown in an outdoor, temperature controlled, underwater tubular photobioreactor. Journal of Biotechnology, 88(3), 239–249. Available from: 10.1016/S0168-1656(01)00280-2 11434969

[mbt214383-bib-0017] Dessì, P. , Rovira‐Alsina, L. , Sánchez, C. , Dinesh, G.K. , Tong, W. , Chatterjee, P. et al. (2021) Microbial electrosynthesis: towards sustainable biorefineries for production of green chemicals from CO_2_ emissions. Biotechnology Advances, 46, 107675. Available from: 10.1016/j.biotechadv.2020.107675 33276075

[mbt214383-bib-0018] Deutzmann, J.S. , Sahin, M. & Spormann, A.M. (2015) Extracellular enzymes facilitate electron uptake in biocorrosion and bioelectrosynthesis. MBio, 6(2), e00496‐15. Available from: 10.1128/mBio.00496-15 25900658 PMC4453541

[mbt214383-bib-0019] Dürre, P. (2017) Gas fermentation—a biotechnological solution for today's challenges. Microbial Biotechnology, 10(1), 14–16. Available from: 10.1111/1751-7915.12431 27790842 PMC5270713

[mbt214383-bib-0020] Eaton, A.D. , Franson, M.A.H. & American Public Health Association . (2005) Standard methods for the examination of water and wastewater. Washington, DC: American Public Health Association.

[mbt214383-bib-0021] Enzmann, F. , Mayer, F. , Stöckl, M. , Mangold, K.M. , Hommel, R. & Holtmann, D. (2019) Transferring bioelectrochemical processes from H‐cells to a scalable bubble column reactor. Chemical Engineering Science, 193, 133–143. Available from: 10.1016/j.ces.2018.08.056

[mbt214383-bib-0022] Ganigué, R. , Puig, S. , Batlle‐Vilanova, P. , Balaguer, M.D. & Colprim, J. (2015) Microbial electrosynthesis of butyrate from carbon dioxide. Chemical Communications, 51(15), 3235–3238. Available from: 10.1039/c4cc10121a 25608945

[mbt214383-bib-0023] Giddings, C.G.S. , Nevin, K.P. , Woodward, T. , Lovley, D.R. & Butler, C.S. (2015) Simplifying microbial electrosynthesis reactor design. Frontiers in Microbiology, 6, 468. Available from: 10.3389/fmicb.2015.00468 26029199 PMC4432714

[mbt214383-bib-0024] Gildemyn, S. , Verbeeck, K. , Slabbinck, R. , Andersen, S.J. , Prévoteau, A. & Rabaey, K. (2015) Integrated production, extraction, and concentration of acetic acid from CO_2_ through microbial electrosynthesis. Environmental Science and Technology Letters, 2(11), 325–328. Available from: 10.1021/acs.estlett.5b00212

[mbt214383-bib-0025] Gottschalk, G. (1986) Bacterial metabolism. New York: Springer. Available from: 10.1007/978-1-4612-1072-6

[mbt214383-bib-0026] Harnisch, F. & Freguia, S. (2012) A basic tutorial on cyclic voltammetry for the investigation of electroactive microbial biofilms. Chemistry, an Asian Journal, 7(3), 466–475. Available from: 10.1002/asia.201100740 22279004

[mbt214383-bib-0027] Harnisch, F. & Holtmann, D. (2017) Electrification of biotechnology: status quo. In: Harnisch, F. & Holtmann, D. (Eds.) Bioelectrosynthesis, Advances in Biochemical Engineering/Biotechnology, vol 167, pp. 1–14. Springer, Cham. Available from: 10.1007/10_2017_41 29209791

[mbt214383-bib-0028] Jourdin, L. , Lu, Y. , Flexer, V. , Keller, J. & Freguia, S. (2016) Biologically induced hydrogen production drives high rate/high efficiency microbial electrosynthesis of acetate from carbon dioxide. ChemElectroChem, 3(4), 581–591. Available from: 10.1002/celc.201500530

[mbt214383-bib-0029] Karthikeyan, R. , Singh, R. & Bose, A. (2019) Microbial electron uptake in microbial electrosynthesis: a mini‐review. Journal of Industrial Microbiology and Biotechnology, 46(9–10), 1419–1426. Available from: 10.1007/s10295-019-02166-6 30923971

[mbt214383-bib-0030] Kim, H.S. (2011) Electrochemical properties of graphite‐based electrodes for redox flow batteries. Bulletin of the Korean Chemical Society, 32(2), 571–575. Available from: 10.5012/bkcs.2011.32.2.571

[mbt214383-bib-0031] Kong, F. , Ren, H.Y. , Pavlostathis, S.G. , Nan, J. , Ren, N.Q. & Wang, A. (2020) Overview of value‐added products bioelectrosynthesized from waste materials in microbial electrosynthesis systems. Renewable and Sustainable Energy Reviews, 125, 109816. Available from: 10.1016/j.rser.2020.109816

[mbt214383-bib-0032] Krieg, T. , Phan, L.M.P. , Wood, J.A. , Sydow, A. , Vassilev, I. , Krömer, J.O. et al. (2018) Characterization of a membrane‐separated and a membrane‐less electrobioreactor for bioelectrochemical syntheses. Biotechnology and Bioengineering, 115(7), 1705–1716. Available from: 10.1002/bit.26600 29578576

[mbt214383-bib-0033] Latif, H. , Zeidan, A.A. , Nielsen, A.T. & Zengler, K. (2014) Trash to treasure: production of biofuels and commodity chemicals via syngas fermenting microorganisms. Current Opinion in Biotechnology, 27, 79–87. Available from: 10.1016/j.copbio.2013.12.001 24863900

[mbt214383-bib-0034] Lee, S.Y. , Oh, Y.K. , Lee, S. , Fitriana, H.N. , Moon, M. , Kim, M.S. et al. (2021) Recent developments and key barriers to microbial CO_2_ electrobiorefinery. Bioresource Technology, 320, 124350. Available from: 10.1016/j.biortech.2020.124350 33186841

[mbt214383-bib-0035] Liu, Z. , Xue, X. , Cai, W. , Cui, K. , Patil, S.A. & Guo, K. (2023) Recent progress on microbial electrosynthesis reactor designs and strategies to enhance the reactor performance. Biochemical Engineering Journal, 190, 108745. Available from: 10.1016/j.bej.2022.108745

[mbt214383-bib-0036] Manchon, C. , Muniesa‐Merino, F. , Llorente, M. & Esteve‐Núñez, A. (2022) Microbial photoelectrosynthesis: feeding purple phototrophic bacteria electricity to produce bacterial biomass. Microbial Biotechnology, 16, 569–578. Available from: 10.1111/1751-7915.14190 36537073 PMC9948228

[mbt214383-bib-0037] Marshall, C.W. , Ross, D.E. , Fichot, E.B. , Norman, R.S. & May, H.D. (2013) Long‐term operation of microbial electrosynthesis systems improves acetate production by autotrophic microbiomes. Environmental Science and Technology, 47(11), 6023–6029. Available from: 10.1021/es400341b 23676111

[mbt214383-bib-0038] May, H.D. , Evans, P.J. & LaBelle, E.V. (2016) The bioelectrosynthesis of acetate. Current Opinion in Biotechnology, 42, 225–233. Available from: 10.1016/j.copbio.2016.09.004 27743996

[mbt214383-bib-0039] McKinlay, J.B. & Harwood, C.S. (2010) Carbon dioxide fixation as a central redox cofactor recycling mechanism in bacteria. Proceedings of the National Academy of Sciences of the United States of America, 107(26), 11669–11675. Available from: 10.1073/pnas.1006175107 20558750 PMC2900684

[mbt214383-bib-0040] Nevin, K.P. , Hensley, S.A. , Franks, A.E. , Summers, Z.M. , Ou, J. , Woodard, T.L. et al. (2011) Electrosynthesis of organic compounds from carbon dioxide is catalyzed by a diversity of acetogenic microorganisms. Applied and Environmental Microbiology, 77(9), 2882–2886. Available from: 10.1128/AEM.02642-10 21378039 PMC3126412

[mbt214383-bib-0041] Nevin, K.P. , Woodard, T.L. , Franks, A.E. , Summers, Z.M. & Lovley, D.R. (2010) Microbial electrosynthesis: feeding microbes electricity to convert carbon dioxide and water to multicarbon extracellular organic compounds. MBio, 1(2), e00103‐10. Available from: 10.1128/mBio.00103-10 20714445 PMC2921159

[mbt214383-bib-0042] Noori, M.T. , Vu, M.T. , Ali, R.B. & Min, B. (2020) Recent advances in cathode materials and configurations for upgrading methane in bioelectrochemical systems integrated with anaerobic digestion. Chemical Engineering Journal, 392, 123689. Available from: 10.1016/j.cej.2019.123689

[mbt214383-bib-0043] Patil, S.A. , Gildemyn, S. , Pant, D. , Zengler, K. , Logan, B.E. & Rabaey, K. (2015) A logical data representation framework for electricity‐driven bioproduction processes. Biotechnology Advances, 33(6), 736–744. Available from: 10.1016/j.biotechadv.2015.03.002 25765230

[mbt214383-bib-0044] Pepè Sciarria, T. , Batlle‐Vilanova, P. , Colombo, B. , Scaglia, B. , Balaguer, M.D. , Colprim, J. et al. (2018) Bio‐electrorecycling of carbon dioxide into bioplastics. Green Chemistry, 20(17), 4058–4066. Available from: 10.1039/c8gc01771a

[mbt214383-bib-0045] Pereira, J.F.S. , Borges, P.H.S. , Moura, G.M. , Gelamo, R.V. , Nossol, E. , Canobre, S.C. et al. (2019) Improved electrochemical performance of pyrolytic graphite paper: electrochemical versus reactive cold‐plasma activation. Electrochemistry Communications, 105, 106497. Available from: 10.1016/j.elecom.2019.106497

[mbt214383-bib-0046] Philips, J. (2020) Extracellular electron uptake by acetogenic bacteria: does H_2_ consumption favor the H_2_ evolution reaction on a cathode or metallic iron? Frontiers in Microbiology, 10, 2997. Available from: 10.3389/fmicb.2019.02997 31998274 PMC6966493

[mbt214383-bib-0060] Prévoteau, A. , Carvajal‐Arroyo, J.M. , Ganigué, R. & Rabaey, K. (2020) Microbial electrosynthesis from CO2: forever a promise? Current Opinion in Biotechnology, 62, 48–57. Available from: 10.1016/j.copbio.2019.08.014 31593911

[mbt214383-bib-0059] Schröder, U. , Harnisch, F. & Angenent, L.T. (2015) Microbial electrochemistry and technology: Terminology and classification. Energy and Environmental Science, 8(2), 513–519. Available from: 10.1039/c4ee03359k

[mbt214383-bib-0047] Shen, N. , Dai, K. , Xia, X.Y. , Zeng, R.J. & Zhang, F. (2018) Conversion of syngas (CO and H_2_) to biochemicals by mixed culture fermentation in mesophilic and thermophilic hollow‐fiber membrane biofilm reactors. Journal of Cleaner Production, 202, 536–542. Available from: 10.1016/j.jclepro.2018.08.162

[mbt214383-bib-0048] Takors, R. , Kopf, M. , Mampel, J. , Bluemke, W. , Blombach, B. , Eikmanns, B. et al. (2018) Using gas mixtures of CO, CO_2_ and H_2_ as microbial substrates: the do's and don'ts of successful technology transfer from laboratory to production scale. Microbial Biotechnology, 11(4), 606–625. Available from: 10.1111/1751-7915.13270 29761637 PMC6011938

[mbt214383-bib-0061] Tejedor‐Sanz, S. & Esteve‐Núñez, A. (2019) *Chapter 18. Fluidized Bed Electrodes in Microbial Electrochemistry*. Microbial Electrochemical Technologies. Boca Ratón, FL: CRC Press, pp. 276–287. Available from: 10.1201/9780429487118

[mbt214383-bib-0049] Tejedor‐Sanz, S. , Fernández Labrador, P. , Manchón, C. & Esteve‐Núñez, A. (2020) Fluidized bed cathodes as suitable electron donors for bacteria to remove nitrogen and produce biohydrogen. Electrochemistry Communications, 116, 106759. Available from: 10.1016/j.elecom.2020.106759

[mbt214383-bib-0050] Tejedor‐Sanz, S. , Fernández‐Labrador, P. , Hart, S. , Torres, C.I. & Esteve‐Núñez, A. (2018) Geobacter dominates the inner layers of a stratified biofilm on a fluidized anode during brewery wastewater treatment. Frontiers in Microbiology, 9, 378. Available from: 10.3389/fmicb.2018.00378 29568284 PMC5853052

[mbt214383-bib-0051] Tejedor‐Sanz, S. , Quejigo, J.R. , Berná, A. & Esteve‐Núñez, A. (2017) Cover picture: the planktonic relationship between fluid‐like electrodes and bacteria: wiring in motion (ChemSusChem 4/2017). ChemSusChem, 10(4), 693–700. Available from: 10.1002/cssc.201700219 27860438

[mbt214383-bib-0052] Tharak, A. & Venkata Mohan, S. (2021) Electrotrophy of biocathodes regulates microbial‐electro‐catalyzation of CO_2_ to fatty acids in single chambered system. Bioresource Technology, 320, 124272. Available from: 10.1016/j.biortech.2020.124272 33142252

[mbt214383-bib-0058] United Nations . (2015) Paris Agreement [WWW Document]. Available from: https://unfccc.int/files/essential_background/convention/application/pdf/english_paris_agreement.pdf

[mbt214383-bib-0053] Vassilev, I. , Dessì, P. , Puig, S. & Kokko, M. (2022) Cathodic biofilms—a prerequisite for microbial electrosynthesis. Bioresource Technology, 348, 126788. Available from: 10.1016/j.biortech.2022.126788 35104648

[mbt214383-bib-0054] Vassilev, I. , Hernandez, P.A. , Batlle‐Vilanova, P. , Freguia, S. , Krömer, J.O. , Keller, J. et al. (2018) Microbial electrosynthesis of isobutyric, butyric, caproic acids, and corresponding alcohols from carbon dioxide. ACS Sustainable Chemistry and Engineering, 6(7), 8485–8493. Available from: 10.1021/acssuschemeng.8b00739

[mbt214383-bib-0055] Vassilev, I. , Kracke, F. , Freguia, S. , Keller, J. , Krömer, J.O. , Ledezma, P. et al. (2019) Microbial electrosynthesis system with dual biocathode arrangement for simultaneous acetogenesis, solventogenesis and carbon chain elongation. Chemical Communications, 55(30), 4351–4354. Available from: 10.1039/c9cc00208a 30911739

[mbt214383-bib-0056] Vilanova, P.B. (2016) Bioelectrochemical transformation of carbon dioxide to target compounds through microbial electrosynthesis . [internal PhD, Universitat de Girona]. Available from: http://hdl.handle.net/10256/13415

[mbt214383-bib-0057] Zhang, T. , Nie, H. , Bain, T.S. , Lu, H. , Cui, M. , Snoeyenbos‐West, O.L. et al. (2013) Improved cathode materials for microbial electrosynthesis. Energy and Environmental Science, 6(1), 217–224. Available from: 10.1039/c2ee23350a

