# Supplementary material for: Novel electrochemical strategies for the microbial conversion of CO2 into biomass and volatile fatty acids using a fluid‐like bed electrode in a three‐phase reactor
Source: Microb Biotechnol. 2024 Jan 17;17(1):e14383. doi: 10.1111/1751-7915.14383 (PMC10832540; doi:10.1111/1751-7915.14383)
Supplement: Supplementary file 1 — Figure S1. [file MBT2-17-e14383-s001.docx]

**SUPPLEMENTARY INFORMATION**


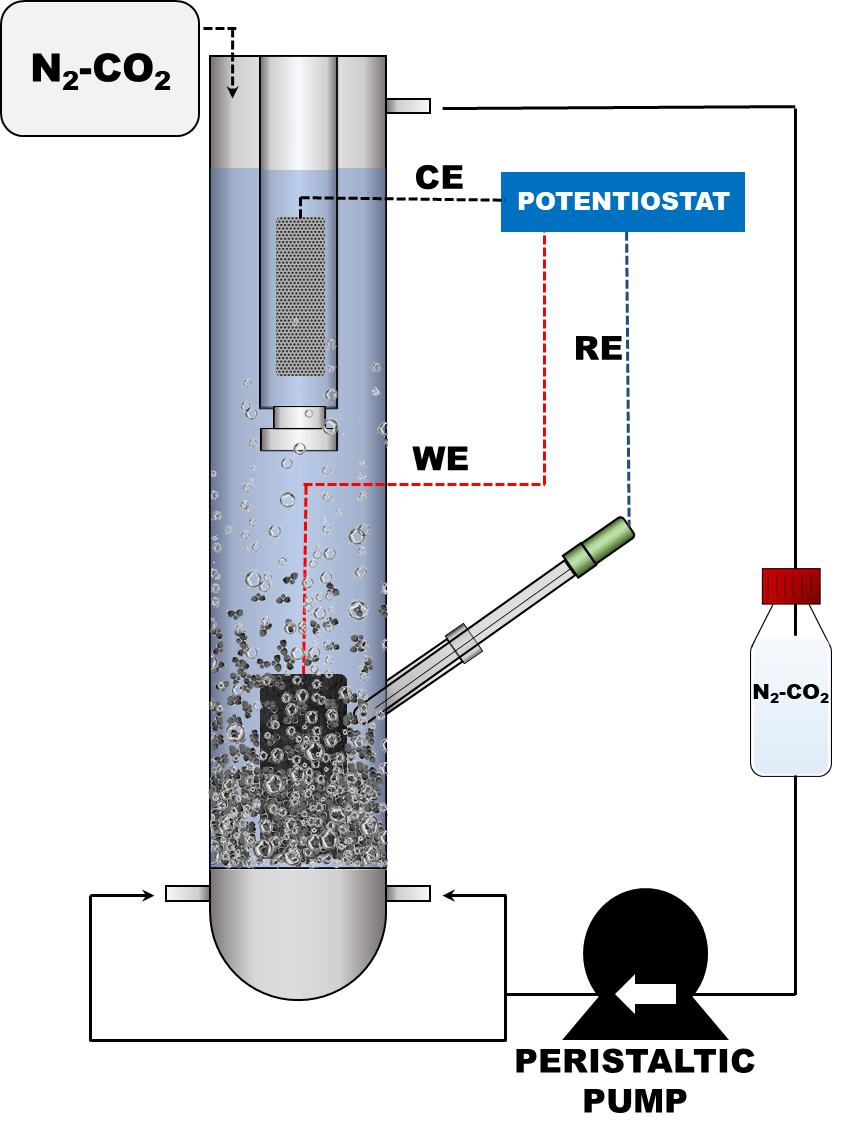


**Figure S1. Reactor configuration and operation setup in phase 2, recirculating the gas from the headspace and operating the system in batch mode.**


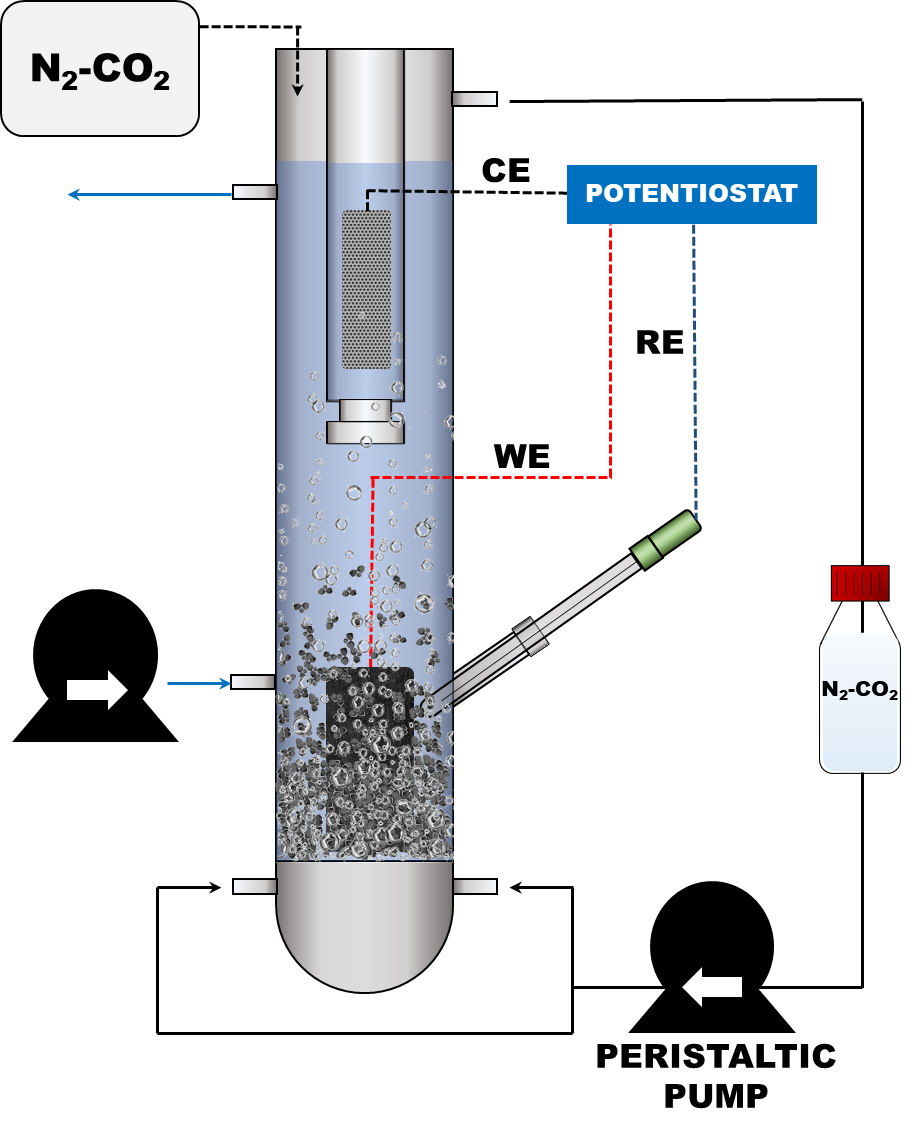


**Figure S2. Reactor configuration and operation setup in phase 3, recirculation the gas from the headspace and operating the system in continuous mode.**

**Abiotic test of resazurin.**

To study the electrochemical behavior of resazurin, a small reactor of 120 mL was built to run abiotic cyclic voltammetries with resazurin (40 µM). The working electrode was a glassy carbon plate (GC) to simulate the electrochemical nature of the glassy carbon bed. Both the current densities and the peaks obtained in presence of resazurin do not correspond to the peaks obtained when the ME-FBR was biologically operated.


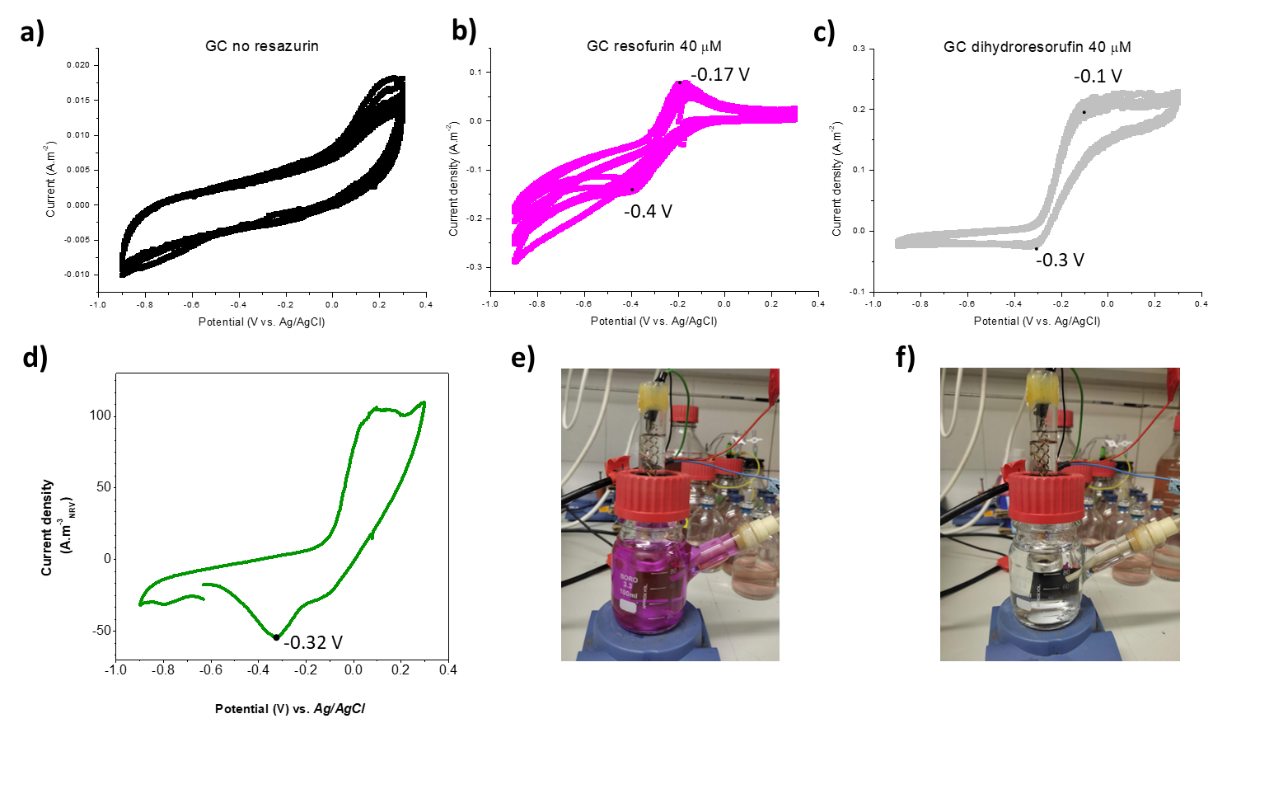


**Figure S3. Abiotic control test run without and with resazurin.** a) Abiotic cyclic voltammetry of the glassy carbon plate. b) Abiotic cyclic voltammetry with resofurin, the reduced form of resazurin. c) Abiotic cyclic voltammetry of dihydroresofurin, the most reduced form of resazurin and can be oxidated to resorufin. d) Cyclic voltammetry of the ME-FBR obtained in phase 3 when the resazurin was added. e) Reactor configuration to perform abiotic cyclic voltammetries with resazurin and f) with dihydroresorufin.

**Calculation on the graphite and electrode surface**

Average size of the granules is 0.2-0.4 mm diameter.

Glassy carbon particles do not present porosity as the commercial characteristics says, “The helium permeability as determined by the vacuum drop method is only 10-11 cm2 s-1”.

Total surface area of the particles is 0.168 m^2^.

The calculations are:

12.5 mL of glassy carbon particles = 10.42 g

100 particles of 0.2-0.4 mm size, weigh 0.0018 g (± 0.0004 g)

10.42 g of glassy carbon / 0.0018 = 578800 particles

The surface area was measured using ImageJ, where 735 particles present an average area of 0.29 mm^2^/ particle.

578800 particles x 0.29 mm^2^/ particles= 0.168 m^2^ is the total surface area of glassy carbon available in the reactor.

Another approximation is to consider the particles as small spheres with an average diameter of 0.3 mm.

4πr2🡪 4 x π x 0.152= 0.283 mm^2^ /particle or sphere, very closed to the surface calculated previously 0.29 mm^2^.
